# Understanding the Contributions of Trait Autism and Anxiety to Extreme Demand Avoidance in the Adult General Population

**DOI:** 10.1007/s10803-022-05469-3

**Published:** 2022-04-18

**Authors:** Rhianna White, Lucy A. Livingston, Emily C. Taylor, Scarlett A. D. Close, Punit Shah, Mitchell J. Callan

**Affiliations:** 1 Department of Psychology, University of Bath, Bath, UK; 2 School of Psychology, Cardiff University, Cardiff, UK; 3 Social, Genetic and Developmental Psychiatry Centre, Institute of Psychiatry, Psychology, London, UK; 4 Neuroscience, King’s College London, London, UK

**Keywords:** Autism, Extreme demand avoidance, Pathological demand avoidance, Anxiety, Adults

## Abstract

Autism and anxiety are thought to be related to extreme demand avoidance (EDA), which is characterised by intense avoidance of everyday demands. However, the relative importance of autism and anxiety to EDA has yet to be investigated, and little is known about EDA in adulthood. We conducted two online survey studies (*Ns* = 267 and 549) with adults in the general population to establish the relative importance of autistic traits and anxiety as predictors of demand avoidance, using dominance analysis. Both autistic traits and anxiety were unique and equally important predictors of demand avoidance. These findings suggest EDA is linked to autism and are consistent with the theory that demand avoidance behaviours are potentially anxiety-driven in adults.

**Supplementary Information:**

The online version contains supplementary material available at 10.1007/s10803-022-05469-3.

Extreme demand avoidance (EDA; also referred to as pathological demand avoidance, or PDA) is characterised by extreme resistance to everyday demands or requests. EDA is a relatively new construct which has seen a recent surge in popularity, especially in the UK. It is not formally recognised as a diagnosis, and as such there are no recognised diagnostic criteria. However, researchers and clinicians have characterised EDA as a set of behaviours in children, including strategic and/or disruptive behaviour to avoid complying, and behaviours typical of labile mood, lack of constraint by social norms, and a heightened need for control (O’Nions et al., 2018). The only prevalence study to date estimated prevalence of autism with EDA or with EDA symptoms at 0.13% in adolescents and adults on the Faroe Islands, compared to a 0.94% prevalence of autism in the same population (Gillberg et al., 2015).

Despite these characterisations, the most appropriate way of conceptualising EDA is debated. Currently, EDA is argued to be part of the autism spectrum, both by researchers and UK charity the PDA Society (PDA Society, 2020). Since autism is now so heterogeneous and the diagnostic criteria have broadened (Timimi & McCabe, 2016), many traits of EDA could potentially be attributed to autism (Kildahl et al., 2021). There are several overlaps between the descriptions of autism and EDA, such as social communication difficulties and a heightened need for control. Malik and Baird (2018) note the similarities between EDA and the restrictive repetitive behaviours of autism. Demand avoidance could be a form of the ‘autistic inertia’ reported by some autistic individuals (Milton, 2019). Despite these overlaps, some practitioners and researchers suggest there are marked differences between EDA and autism which indicate that EDA is a separate condition (e.g. O’Nions, Viding, et al., 2014). Persons with EDA are thought to have higher social fluency, engage in role play, and utilise socially strategic manipulative behaviour. Alternatively, as Woods (2019) argues, perhaps EDA is best described as a behaviour profile that can be seen across a variety of conditions (such as, potentially, conduct disorder and ADHD); or perhaps it can be attributed to (a combination of) already recognised conditions (Green et al., 2018).

EDA is believed to be driven and maintained by anxiety (e.g. O’Nions & Eaton, 2020). Indeed, some argue that demand avoidance may be described as a rational method of avoiding anxiety, especially for those with limited autonomy, such as children (Moore, 2020; Woods, 2019). Demand avoidance behaviours could be described as learned coping mechanisms, developed in response to extreme anxiety caused by an aversive stimulus. In this framework, avoiding or delaying the imposed demand of an aversive activity enables a person to regain control of the situation, thereby reducing anxiety. This reinforces the use of avoidance behaviours in response to demands. EDA may therefore have some features in common with maladaptive coping mechanisms, such as eating disorders, selective mutism, and self-harm. Adults with an EDA profile have reported high levels of anxiety (“My primary emotion is anxiety”), and experience a lack of control as catastrophic (“If I feel out of control everything goes very wrong, very quickly”, (Cat, 2018, p. 57, p. 107). Avoiding demands is itself likely to increase anxiety in the long-term, as found in research into procrastination (Abbasi & Alghamdi, 2015 for a review), thus setting up a self-perpetuating (and possibly amplifying) cycle of anxiety and avoidance. Avoidance may be initially localised to aversive demands, but then spread to, or ‘contaminate’ neutral or even positive tasks. Stuart et al. (2020) found anxiety (and especially intolerance of uncertainty, an underlying construct thought to lead to some of the principal symptoms of anxiety) was associated with demand avoidance behaviours in children when controlling for an autism diagnosis. Autism is itself associated with anxiety, with a recent meta-analysis estimating lifetime prevalence of any anxiety disorder at 42% for autistic adults (Hollocks et al., 2019), which must be taken into account when exploring the relationships between autism, anxiety, and EDA.

EDA is believed to emerge in childhood and continue into adulthood (Egan et al., 2019; Newson et al., 2003). Newson et al. originally described EDA in a position paper, based on experiences at a specialist clinic assessing children with supposed ‘atypical’ autism. The authors followed up with 18 young people who had been assessed as children, all of whom remained highly demand avoidant in adulthood, although some had become less avoidant. The continuation of EDA into adulthood is supported by recent anecdotal evidence from clinicians. However, EDA traits are thought to decrease as a child gets older (O’Nions, Christie, et al., 2014; Stuart et al., 2020). Reduction of EDA traits with age may be due to a child’s improvement in skills such as self-regulation and communication and their ability to self-advocate. It is possible that this trajectory of diminishing demand avoidance behaviours continues throughout adulthood, although Egan et al. (2019, 2020) have found no relationship between EDA traits and age in adults. Similarly, anxiety disorders in the general population have been found to decrease with age (Scott et al., 2008), so this may potentially also have an effect in reducing demand avoidance behaviours, if indeed anxiety drives demand avoidance.

A final consideration when investigating links to EDA is participant sex. In its original conception, EDA was thought to have an equal sex ratio (Newson et al., 2003), which is supported by some recent studies finding no gender difference in EDA trait scores (O’Nions, Christie, et al., 2014; Stuart et al., 2020). It has been suggested that EDA is an example of a more typically female autism profile (O’Nions, Happé, et al., 2016), in which case a more balanced sex-ratio would be expected, compared to that typically seen in autism, which is around 3:1 males to females (Loomes et al., 2017). However, this even sex-ratio in EDA is not a consistent finding, with other studies finding higher demand avoidance traits in women (Egan et al., 2020), or, conversely, higher traits in men (Egan et al., 2019). If EDA is indeed a form of autism, it would seem likely that it would have a similar sex ratio to autism. Given these gaps in knowledge about the associations between sex and age and EDA, it is important to factor age and sex into investigations of demand avoidance traits.

As the foregoing summarises, it is debated whether EDA falls on the autism spectrum, or whether it may be better described as a behaviour profile driven by anxiety. Understanding whether EDA is more closely linked to autism or anxiety will help address this issue, which has never been empirically tested in adults. Very few studies have examined demand avoidance traits in the adult population despite the fact that EDA is believed to continue into adulthood. The current paper aims to build on previous research (Egan et al., 2019; Stuart et al., 2020) by investigating the relative importance of autistic traits and anxiety when predicting demand avoidance traits in the adult general population. We conducted two studies, with participants completing measures of mental health symptoms (anxiety, depression, and stress), autistic traits, and demand avoidance traits. We followed the approach of Egan et al. (2019, 2020) in measuring demand avoidance traits in the general population rather than persons with an identified EDA profile, given the lack of clear diagnostic criteria for EDA. We included both autistic traits and anxiety in all models to account for confound due to the known association between autism and anxiety, and accounted for depression and stress to control for emotional symptoms other than anxiety. Based on the existing literature, we predicted that autistic traits and anxiety would both be associated with demand avoidance traits.

## Study 1

### Method

UK residents were recruited online through Amazon’s Mechanical Turk and supplemented with participants from a UK undergraduate student population (total *N*=267; 64% female; *M*_age_ = 32.7 years, *SD*_age_ = 13.4 years). The Mechanical Turk participants had completed at least 50 tasks and had an approval rate greater than or equal to 95% on the platform. Sample characteristics and scale reliabilities are shown in Table 1. One participant who did not identify as male or female was excluded from analyses where sex was a variable. Participants completed three questionnaires which were presented in a randomised order and followed by questions about age and sex, as assigned at birth. All studies were approved by the University of Bath ethics committee. Participants in both studies were paid for their time, according to the standard rates of the recruitment sites, and provided electronic consent. The student participants in Study 1 received course credits. Both studies were conducted using Qualtrics to collect data. The EDA community was not involved in the development of studies 1 or 2.

**Table 1:** Descriptive statistics and correlations (N = 266)

Measure	*M* (*SD*)	1.	2.	3.	4.	5.	6.
1. Autistic traits (AQ-10)	20.24 (4.39)	(0.69)					
2. Depression (DASS-D)	11.36 (10.50)	0.36***	(0.93)				
3. Anxiety (DASS-A)	9.11 (8.88)	0.36***	0.76***	(0.88)			
4. Stress (DASS-S)	13.28 (8.94)	0.32***	0.77***	0.77***	(0.89)		
5. Demand avoidance (EDA-QA)	43.41 (12.76)	0.40***	0.59***	0.69***	0.56***	(0.93)	
6. Age	32.72 (13.40)	-0.16**	-0.15*	-0.15*	-0.07	-0.15*	
7. Sex (0=Female, 1=Male)	65% female	0.24***	0.08	0.01	-0.02	0.24***	0.02

*Note*. EDA-QA, Extreme Demand Avoidance Questionnaire – Adult version; AQ-10, brief Autism Spectrum Quotient; DASS, Depression, Anxiety and Stress Scale. Standardised Cronbach’s alpha reliabilities are presented along the diagonal

Demand avoidance traits were assessed using the Extreme Demand Avoidance Questionnaire – Adult version (EDA-QA; Egan et al., 2019), a 26-item measure assessing self-reported EDA behaviours (e.g. ‘I sometimes use outrageous or shocking behaviour to get out of doing something,’ and ‘I obsessively resist and avoid ordinary demands and requests.’). The EDA-QA is scored on a 4-point scale (1 = *Not true*, to 4 = *Very true*), with higher scores indicating more EDA traits.

Autistic traits were assessed with the brief 10-item Autism Spectrum Quotient (AQ-10, Allison et al., 2012), e.g. ‘I find it easy to ‘read between the lines’ when someone is talking to me,’ and ‘I find it easy to work out what someone is thinking or feeling just by looking at their face.’ Items are answered on a 4-point scale (1 = *Definitely agree*, to 4 = *Definitely disagree*), with higher scores indicating more autistic traits. Binary scoring is typically used for the AQ-10, but we chose to score items 1–4 in order to retain more detail in responses and increase variability in scores, as recommended by Stevenson and Hart (2017). The scale therefore had a range of 10–40, with higher scores indicating more autistic traits.

Depression, anxiety, and stress symptoms were measured using the three subscales of the Depression, Anxiety and Stress Scale – 21 Items (DASS-21; Lovibond & Lovibond, 1995). The DASS-21 is scored on a 4-point scale indicating how much the items applied over the past week (0 = *Did not apply to me at all*, to 3 = *Applied to me very much or most of the time*), with higher scores on each subscale indicating higher levels of depression, anxiety, or stress. Example questions include ‘I felt that I had nothing to look forward to,’ ‘I felt scared without any good reason,’ and ‘I found myself getting agitated,’ for the depression, anxiety, and stress scales respectively. Scores on the DASS-21 were doubled so they were comparable to the full 42-item DASS. Non-clinical norms for the DASS-21 have previously been established at 5.66 for depression, 3.76 for anxiety, and 9.46 for stress (Henry & Crawford, 2005), with ‘severe’ thresholds of 21, 15, and 26 for depression, anxiety, and stress respectively.

## Results

All analyses were performed in R (version 3.6.1). Descriptive statistics and Pearson correlations between all measures are displayed in Table 1. Demand avoidance correlated significantly with all variables, including autistic traits and anxiety. As expected, autistic traits correlated positively with anxiety.

Multiple linear regression was conducted with autistic traits, depression, anxiety, stress, sex, and age as predictors of demand avoidance traits (Table 2). Autistic traits and anxiety were both significant predictors, with more autistic traits and higher anxiety uniquely predicting higher demand avoidance traits (Table 2). Being male also predicted higher demand avoidance. As a sensitivity analysis, we used structural equation modelling (SEM) to model this regression whilst accounting for measurement error. This method is recommended by Westfall and Yarkoni (2016), who showed that the Type I error rate increases when testing incremental validity without modelling measurement error. We therefore supplemented our multiple regression analyses with weighted least squares SEM, to determine whether measurement error was affecting the pattern of results. SEM analysis confirmed that autistic traits, anxiety, and sex remained significant predictors of demand avoidance when modelling measurement error (see Supplementary Table 1 for full SEM results).

**Table 2:** Multiple regression predicting demand avoidance in Study 1 (N = 266)

Predictors	*B* [95% CI]	*SE*	*t*	*b*	*p*
Autistic traits	0.29 [0.02, 0.55]	0.14	2.10	0.10	0.037
Depression	0.08 [-0.10, 0.25]	0.09	0.84	0.06	0.400
Anxiety	0.81 [0.61, 1.02]	0.10	7.80	0.57	<0.001
Stress	0.07 [-0.14, 0.28]	0.11	0.66	0.05	0.510
Age	-0.03 [-0.11, 0.05]	0.04	-0.81	-0.03	0.417
Sex (0=Female, 1=Male)	5.59 [3.29, 7.88]	1.16	4.80	0.21	<0.001

*R* ^2^_adj_=0.54, *F*(6, 259)= 52.87, *p*<.001

### Dominance Analysis

We used dominance analysis to examine the relative importance of autistic traits and anxiety as putative explanatory variables for demand avoidance traits. Comparing the sizes of regression coefficients is insufficient for determining relative importance, as coefficients are affected by correlations between predictors. Dominance analysis evaluates the relative contribution each predictor in a regression model makes to the prediction of the outcome by computing the semi-partial correlation squared (*sr* ^*2*^) of the predictor across all possible regression subset models. We used general dominance weights to assess predictor importance; these weights are the average of the predictor’s incremental contribution across all possible subset models. General dominance weights sum to the total *R* ^2^ for the model. Predictors can therefore be ranked by their general dominance weights to determine their relative importance to the outcome variable. Bootstrapping is then used to test the stability of these dominance relationships.

We followed the methods described by Braun et al. (2019) to account for sampling and measurement error while performing dominance analysis, using the domWeightTool in R with 1,000 simulated runs. General dominance relationships were explored across correlation matrices, uncorrected and corrected for measurement error (corrected matrix in Table 3). These corrections were made based on the observed standardised Cronbach’s alphas of the measures. We assumed perfect reliability for age and sex. The dominance analyses were set up in the same way as the multiple regression, with autistic traits, depression, anxiety, stress, age, and sex predicting demand avoidance.

**Table 3:** General dominance analysis weight and rank values for corrected correlation matrix

	Corrected matrix	
Predictors	Mean dominance (*SD*)	Sig.
Dominance weights		
Anxiety	0.286 (0.050)	100%
Depression	0.126 (0.021)	100%
Stress	0.124 (0.021)	100%
Autistic traits	0.081 (0.024)	97%
Sex	0.049 (0.020)	67%
Age	0.009 (0.006)	2%
R^2^	0.682 (0.068)	
Dominance ranks		
Anxiety	1.000 (0.000)	
Depression	2.579 (0.628)	
Stress	2.645 (0.624)	
Autistic traits	3.946 (0.682)	
Sex	4.849 (0.480)	
Age	6.464 (0.513)	

*Note*. Sig. = percentage of runs where predictor was significantly different from zero

Anxiety was dominant over all other predictors, ranking first in the corrected matrix with a much higher general dominance weight (Table 3). Table 4, which shows the percentage of runs in which two predictors were found to be significantly different from one another, confirms this result. In 99% of the simulated runs anxiety was found to be significantly different from autistic traits as a predictor of demand avoidance traits. This suggests that the dominance of anxiety over autistic traits is highly replicable.

**Table 4:** Significant differences between predictors for corrected correlation matrix

	Autistic traits	Depression	Anxiety	Stress	Age
Depression	53%				
Anxiety	99%	99%			
Stress	51%	19%	100%		
Age	97%	100%	100%	100%	
Sex	35%	79%	100%	80%	65%

*Note.* Values are percentages of 1,000 runs with significant differences in dominance within each pair of predictors

Depression and stress (while not significant predictors from the multiple regression analysis) also tended to be dominant over autistic traits. However, Table 4 suggests this result is not especially reliable. Depression and stress were dominant over autistic traits in only 53% and 51% of runs respectively for the corrected matrix. Anxiety was dominant over both depression and stress, suggesting that the link between anxiety and demand avoidance is specifically due to anxiety rather than co-occurring depression or stress.

## Discussion

The results of Study 1 suggest that autistic traits were a weaker unique predictor of demand avoidance traits than anxiety, and weaker than might be expected, given previous research (e.g. Egan et al., 2019). Autistic traits were a significant predictor of EDA traits, but was shown in the dominance analysis to be a weaker predictor than depression and stress, both of which were non-significant in the multiple regression analysis. Men reported significantly higher demand avoidance than women, but the dominance analysis, which is sensitive to intercorrelations between predictors, showed sex was not an especially useful predictor of demand avoidance. Critically, anxiety was the most important predictor of demand avoidance.

The sample size for Study 1 was comparatively small, so we chose to replicate the effects in a larger sample. While useful for the purposes of Study 1, the AQ-10 is an abbreviated version of the full Autism Spectrum Quotient (AQ; Baron-Cohen et al., 2001), and does not capture as much of the domain space of autistic traits. For these reasons, Study 2 was conducted to clarify the results of Study 1 using an independent larger sample and the full, more comprehensive, AQ measure.

## Study 2

### Method

UK-based participants (*N*=549) were recruited through Prolific. The sample was 49% female, aged from 18 to 67 years (*M*_*age*_=35.5 years, *SD*_*age*_ = 11.8 years). Sample characteristics and scale reliabilities are given in Table 5. One participant who did not identify as male or female was excluded from analyses where sex was a variable. The same procedure was used as in Study 1, with participants completing an online survey consisting of three questionnaires, with data collected from May 11th to 12th 2020. Participants failing an attention check were excluded. Pre-screening filters were used to ensure all participants were currently living in the UK and spoke English as a first language.

**Table 5:** Descriptive statistics and correlations (N = 548)

Measure	*M* (*SD*)	1.	2.	3.	4.	5.	6.	7.
1. Autistic traits (AQ-50)	114.47 (16.59)	(0.88)						
2. Depression (DASS-D)	13.30 (10.22)	0.32***	(0.92)					
3. Anxiety (DASS-A)	6.89 (7.76)	0.35***	0.61***	(0.86)				
4. Stress (DASS-S)	13.92 (9.07)	0.35***	0.68***	0.70***	(0.87)			
5. Demand avoidance (EDA-QA)	42.88 (9.31)	0.47***	0.40***	0.52***	0.46***	(0.87)		
6. COVID-19 concern	3.26 (1.09)	0.13**	0.21***	0.27***	0.28***	0.09*		
7. Age	35.45 (11.77)	-0.05	-0.18***	-0.20***	-0.15***	-0.26***	0.09*	
8. Sex (0=Female, 1=Male)	49% female	0.05	0.04	0.02	0.00	0.14***	-0.19***	-0.04

*Note*. EDA-QA, Extreme Demand Avoidance Questionnaire – Adult version; AQ-50, full Autism Spectrum Quotient; DASS, Depression, Anxiety and Stress Scale. Standardised Cronbach’s alpha reliabilities are presented along the diagonal

Demand avoidance traits, depression, anxiety, and stress were measured using the EDA-QA and DASS-21 as before. Following the results of Study 1, the full Autism Spectrum Quotient (AQ-50; Baron-Cohen et al., 2001) was used to measure autistic traits. The AQ-50 has acceptable internal consistency and test-retest reliability in the general population (e.g. Stevenson & Hart, 2017). As before, we chose to score items 1–4 in order to retain more detail in responses and increase variability in scores, as recommended by Stevenson and Hart. The scale therefore had a range of 50–200, with higher scores indicating more autistic traits.

Given that Study 2, unlike Study 1, was conducted during the COVID-19 pandemic, we tested whether concern around COVID-19 might modulate the relationships between our predictors and EDA, given the increased prevalence of mental health problems in the UK during the pandemic onset (Shevlin et al., 2020). We asked participants ‘How concerned do you feel about COVID-19?’ which was answered on a 5-point scale from *Not at all concerned* to *Extremely concerned* (Nelson et al., 2020).

## Results

Descriptive statistics and Pearson correlations are displayed in Table 5. There was a similar pattern of correlations to Study 1, with demand avoidance significantly correlating with all variables of interest, most strongly with autistic traits and anxiety.

Table 6 displays the results of a multiple regression with autistic traits, depression, anxiety, stress, age, and sex predicting demand avoidance traits. Higher autistic traits and higher anxiety and stress significantly predicted higher demand avoidance traits, as did being male and being younger. As in Study 1, SEM was used to test whether measurement error affected the results. The pattern of results was the same (see Supplementary Table 2 for full results).

**Table 6:** Multiple regression predicting demand avoidance in Study 2 (N=548)

Predictor	*B* [95% CI]	*SE*	*t*	*b*	*p*
Autistic traits	0.17 [0.13, 0.21]	0.02	8.59	0.31	<0.001
Depression	0.02 [-0.07, 0.10]	0.04	0.38	0.02	0.701
Anxiety	0.35 [0.23, 0.46]	0.06	5.97	0.29	<0.001
Stress	0.12 [0.02, 0.22]	0.05	2.26	0.12	0.024
Age	-0.13 [-0.18, -0.08]	0.03	-4.83	-0.16	<0.001
Sex (0=Female, 1=Male)	2.19 [0.98, 3.39]	0.61	3.57	0.12	<0.001

*R* ^2^_adj_=0.41, *F(*6, 541)=64.26, *p*<.001

We fitted an additional multiple regression model, including interactions between participants’ level of concern about COVID-19 and each other variable in the regression model. Analyses revealed that degree of concern did not uniquely predict EDA or moderate any of the relationships between other predictors and EDA (see Supplementary Table 3 for full results).

### Dominance Analysis

As in Study 1, we performed dominance analysis to test the relative importance of predictors of demand avoidance traits, using 1000 simulated runs with the domWeightTool. The corrected matrix in Table 7 shows that both autistic traits and anxiety hold first rank for general dominance weight, with anxiety ranked dominant over autistic traits. However, this difference in dominance is only true in 25% of simulated runs (Table 8), suggesting that this difference is not reliable and both autistic traits and anxiety are comparable in terms of importance in predicting demand avoidance.

**Table 7:** General dominance analysis weight and rank values for corrected correlation matrix

	Corrected matrix	
Predictors	Mean dominance (*SD*)	Sig.
Dominance weights		
Autistic traits	0.156 (0.031)	100%
Anxiety	0.155 (0.027)	100%
Stress	0.092 (0.018)	100%
Depression	0.057 (0.011)	100%
Age	0.042 (0.012)	90%
Sex	0.020 (0.010)	38%
R^2^	0.526 (0.038)	
Dominance ranks		
Anxiety	1.519 (0.533)	
Autistic traits	1.544 (0.582)	
Stress	2.961 (0.293)	
Depression	4.180 (0.438)	
Age	4.910 (0.546)	
Sex	5.937 (0.421)	

*Note*. Sig. = percentage of runs where predictor was significantly different from zero

**Table 8:** Significant differences between predictors for corrected correlation matrix

	Autistic traits	Depression	Anxiety	Stress	Age
Depression	96%				
Anxiety	25%	99%			
Stress	66%	81%	82%		
Age	98%	22%	100%	74%	
Sex	100%	74%	100%	97%	35%

*Note.* Values are percentages of 1,000 runs with significant differences in dominance within each pair of predictors

As in Study 1, stress also predicts demand avoidance, but to a weaker degree than autistic traits and anxiety. Despite sex being a highly significant predictor in the multiple regression analysis, sex is only significantly different from zero in 38% of simulated runs in the dominance analysis (Table 7), suggesting sex is not an especially important predictor of EDA compared to autistic traits and anxiety. The full corrected model accounts for approximately 53% of variance (*R* ^*2*^=0.53).

### Discussion

We conducted two studies to examine the relative importance of autistic traits and anxiety in relation to demand avoidance traits in the adult general population. In Study 1, anxiety was the most important predictor of demand avoidance traits, over and above autistic traits. The results of Study 2, however, suggest that autistic traits and anxiety are comparably important when predicting demand avoidance. Taken together, these results suggest both autism and anxiety are important factors contributing to EDA.

These studies support the previous research of Stuart et al. (2020), which linked anxiety and EDA in children. By accounting for depression and stress, the current study demonstrates there is a link between EDA and anxiety rather than with general emotional symptoms, which agrees with the heightened anxiety reported in personal and clinical accounts. Anxiety has been posited as the driver behind demand avoidance behaviours (e.g. O’Nions & Eaton, 2020). It is, however, unclear whether anxiety or demand avoidance arises first, since they are likely to be interlinked and self-reinforcing. Now we have firmly established a link between anxiety and EDA in adults, moving forward it would be useful to test whether there are specific components, or types, of anxiety are associated with EDA. For instance, whether social anxiety particularly provokes demand avoidance behaviours, given that demands are principally made by other people; or whether the anxiety response is particularly driven by intolerance of uncertainty, as Stuart et al. suggest. The current study used a cross-sectional method of assessing links between anxiety and autism but it would be useful to look at this relationship longitudinally, perhaps by experience sampling, to test the theory that high anxiety is a direct driver of demand avoidance behaviours.

We replicated previous research linking autism and EDA in adults (Egan et al., 2019). Autistic traits predicted demand avoidance traits when accounting for the potentially confounding factor of anxiety. This suggests that autism makes a unique contribution to predicting EDA, consistent with the prevailing theory that EDA is a part of the autism spectrum. Currently, there does not seem to be clear rationale to make EDA a separate sub-division of autism, given the removal of clinical subtypes such as Asperger’s syndrome from the DSM-5 (Green et al., 2018). However, anecdotally, clinicians who recognise EDA report that they find a clear distinction between autism and EDA (e.g. Eaton et al., 2018). In practice, it seems that clinicians are identifying a difference between the two which does not seem to be translating into research.

In our studies, men had more demand avoidance traits, as in Egan et al. (2019). However, our results suggest that this sex difference is of relatively low importance when considering factors associated with demand avoidance traits. We also found a link between increasing age and fewer demand avoidance traits. This suggests that the pattern of reduction in demand avoidance with age seen in childhood continues into adulthood. Development of self-regulation skills and emotional maturation continues well into adulthood, with adults generally becoming more competent at self-management with time (Denissen et al., 2013). However, the impact of increasing age was much less important than the effect of higher autistic traits and increased anxiety.

We focused on the links between EDA, autism, and anxiety, but it would be interesting to investigate the associations between EDA and oppositional defiant disorder (ODD). Some have suggested that EDA can be described as ODD (e.g. Malik & Baird, 2018), which is characterised by a longstanding pattern of angry or irritable mood, vindictiveness, and argumentative or defiant behaviour, including noncompliance with rules and requests from authority figures. The avoidance behaviours seen in EDA could therefore be similar to the noncompliance seen in ODD. However, EDA is thought to differ from ODD in that people with EDA are ‘non-compliant’ in response to very simple demands, may lack the image maintenance seen in ODD, and since incentives are ineffectual in reducing avoidance (O’Nions, Happé, et al., 2016). The validity of ODD itself as a condition is also under question, given the debate around the ethics of medicalising deviance (e.g. Bosk, 2013).

The current conception of EDA is heavily based on the accounts presented by Newson et al. (2003) and the validity of this account needs to be tested further. Some of the features have been revised, for example O’Nions, Gould, et al., (2016) did not find evidence of the stronger imaginative ability Newson et al. described in EDA compared to autism. More generally, the authors found that many features of EDA were in fact commonly found in autistic children (e.g., uncooperativeness, labile mood, blaming others). However, the authors identified several features they believed to be EDA-specific (e.g., ‘manipulative’ and socially shocking behaviour).

There are several limitations of the current study. One potential limitation is that Study 2 was carried out during the COVID-19 pandemic, which may have influenced the mental health symptoms of participants, and possibly their demand avoidance behaviours. Our results show no evidence of this association. Nonetheless, it is is possible that the higher-than-average rates of anxiety, depression, and stress symptoms reported by our participants may limit the generalisability of our results to the wider population. Our sample may also be enriched for EDA traits, so our results may again be less generalisable, and it would therefore be beneficial to use an epidemiological sample in a future study, although this data is not currently available. We tested associations with demand avoidance behaviours in the general population, and an important next step would be to repeat these analyses in population-representative clinical samples. This should hopefully reduce the uncertainty around the relative importance of the association between anxiety and EDA compared to autistic traits.

It is possible that the difference between the results of Studies 1 and 2 was due partly to the smaller sample size and use of a short and less reliable measure of autistic traits in Study 1. The AQ-50 used in Study 2 may simply be measuring autism more accurately than the AQ-10. Alternatively, the longer measure of autistic traits may contain more conceptual overlaps with the EDA-QA measure, which may partly explain the link found between EDA and autism. The link found between autism and EDA in the current study may in part be related to the measure used to detect demand avoidance traits. More specific tools for testing EDA traits are needed which avoid, as far as possible, overlaps with recognised conditions such as autism and ODD. It would be useful to streamline the EDA-QA, and possibly reduce conceptual overlaps with ODD, by extending the current studies to look at oppositional defiant traits.

Our research supports the use of anxiety-reducing approaches for managing demand avoidance behaviours in adults with EDA, both in terms of professionals working with adults and adults themselves managing their demand avoidance. Despite the links between autism and EDA found in this study, previous work suggests that approaches recommended for autism (such as establishing routine and structure) can be counterproductive in cases of clear EDA (Christie, 2007). O’Nions and Eaton (2020) recommend a ‘low-demand, low-arousal approach’ with children, and this may also be a useful starting point for adults with EDA.

## Electronic Supplementary Material

Below is the link to the electronic supplementary material.

Supplementary Material 1
