# Supplementary material for: Understanding the Contributions of Trait Autism and Anxiety to Extreme Demand Avoidance in the Adult General Population
Source: J Autism Dev Disord. 2022 Apr 18;53(7):2680–8. doi: 10.1007/s10803-022-05469-3 (PMC9015283; doi:10.1007/s10803-022-05469-3)
Supplement: Supplementary file 1 — Supplementary Material 1 [file 10803_2022_5469_MOESM1_ESM.docx]

**Supplementary Material**

**Structural Equation Modelling**

Following Westfall and Yarkoni’s (2016) recommendations for testing incremental validity, we modelled autistic traits, anxiety, depression, stress, age, and sex as predictors of demand avoidance traits using a structural equation model (SEM). Standard multiple regression, as we reported in the main article, does not account for measurement error, which may inflate the likelihood of committing Type I errors. SEM accounts for measurement error by using latent variables generated from individual indicators rather than sum scores.

SEM analyses were performed using the lavaan package (Rosseel, 2012) in R (version 3.6.1) for both Studies 1 and 2. Diagonally weighted least squares for means and variances estimation was used because the EDA-QA uses a 4-point Likert scale and thus is arguably measured ordinally. Latent constructs for autistic traits, anxiety, depression, stress, and extreme demand avoidance traits were derived from the AQ-10/50, separate subscales of the 21-item Depression, Anxiety, and Stress Scale, and the EDA-QA, respectively. We assumed that age and sex were measured with perfect reliability. The structural model was constructed as a multiple regression (as in the main article), with autistic traits, depression, anxiety, stress, age, and sex predicting demand avoidance traits. We specified correlations between the error variances for the two reversed worded items (EDA14 and EDA20) in these analyses (see Brown & Moore, 2012).

As shown in Supplementary Tables 1 and 2, for both studies, latent autistic traits and anxiety accounted for unique variance in EDA traits in the expected directions. Thus, these analyses yielded the same conclusions as those we report in the main article from standard multiple regression analyses. The one discrepancy of note is that age is a significant predictor in the Study 1 SEM, and was non-significant in the multiple regression analysis reported in the paper.

**Study 1 SEM**

Supplementary Table 1

*Regressions Predicting Demand Avoidance Traits in Structural Equation Model from Study 1 (N=266)*

| Predictor | *B* | *SE* | *z* | *p* | *b* |
| --- | --- | --- | --- | --- | --- |
| Autistic traits | 0.62 | 0.20 | 3.17 | .002 | .28 |
| Depression | -0.12 | 0.11 | -1.06 | .29 | -.10 |
| Anxiety | 0.78 | 0.20 | 3.95 | <.001 | .48 |
| Stress | 0.23 | 0.17 | 1.31 | .19 | .15 |
| Age | -0.01 | 0.004 | -3.05 | .002 | -.21 |
| Male | 0.41 | 0.11 | 3.66 | <.001 | .23 |
| Robust fit statistics χ^2^ (1640, N=266) = 2741.33, p<.001; RMSEA=0.05 (90% CI: 0.047, 0.054), CFI=0.85. *B* and *b* indicate unstandardised and standardised coefficients respectively. | | | | | |

**Figure 1**

Study 1 SEM Structural Path Diagram

**
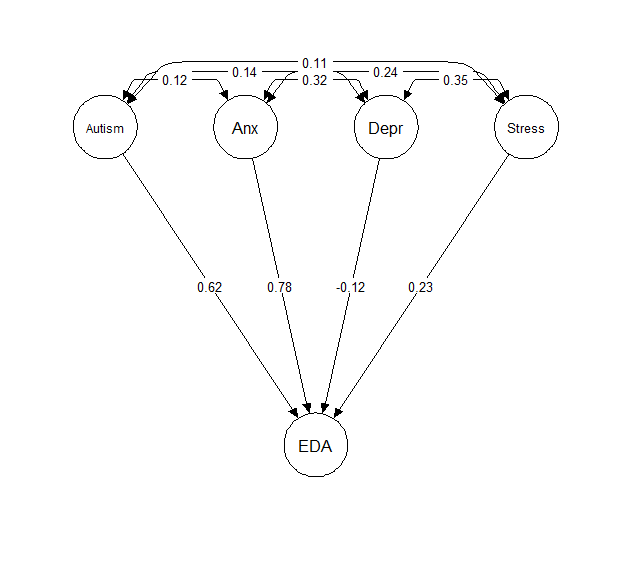
**

*Note.* Coefficients are unstandardized. All manifest variables (including age and sex) omitted for clarity.

**Study 2 SEM**

Supplementary Table 2

*Regressions Predicting Demand Avoidance Traits in Structural Equation Model from Study 2 (N=548)*

| Predictor | *B* | *SE* | *z* | *p* | *b* |
| --- | --- | --- | --- | --- | --- |
| Autistic traits | 0.95 | 0.19 | 5.10 | <.001 | .38 |
| Depression | -0.03 | 0.05 | -0.55 | .58 | -.03 |
| Anxiety | 0.27 | 0.09 | 3.00 | .003 | .20 |
| Stress | 0.28 | 0.09 | 3.13 | .002 | .24 |
| Age | -0.02 | 0.003 | -6.56 | <.001 | -.31 |
| Male | 0.17 | 0.06 | 2.91 | .004 | .13 |
| Robust fit statistics χ^2^ (4741, N=548) = 8831.77, p<.001; RMSEA=0.04 (90% CI: 0.038, 0.041), CFI = 0.66. *B* and *b* indicate unstandardised and standardised coefficients respectively. | | | | | |

**Figure 2**

Study 2 SEM Structural Path Diagram**
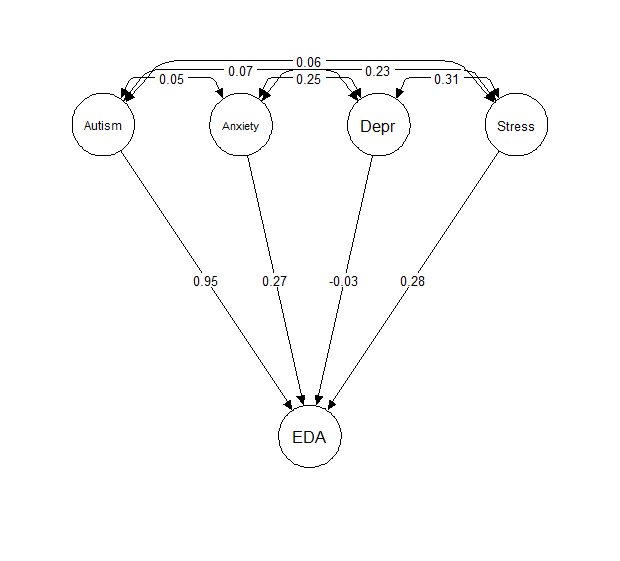
**

*Note.* Coefficients are unstandardized. All manifest variables (including age and sex) omitted for clarity.

**COVID-19 Concern**

Given that Study 2, unlike Study 1, was conducted during the COVID-19 pandemic, it was possible that participants may have been experiencing more anxiety, depression, and stress than typically. We asked participants ‘How concerned do you feel about COVID-19?’ which was answered on a 5-point scale from ‘Not at all concerned’ to ‘Extremely concerned’ (Nelson et al., 2020).

To test for any effects of COVID-19 concern, we ran a multiple regression model as in Study 2, but including participants’ concern about COVID-19 and interactions between COVID-19 concern and each other variable in the model (i.e. autistic traits, depression, anxiety, stress, age, and sex). The results of this regression are shown in Supplementary Table 3.

Supplementary Table 3

*Regression Results Predicting Demand Avoidance Traits in Study 2 (N=548)*

| Predictors | *B* | *SE* | *t* | *p* |
| --- | --- | --- | --- | --- |
| Autistic traits | 0.17 | 0.02 | 8.26 | <.001 |
| Depression | 0.01 | 0.04 | 0.31 | .75 |
| Anxiety | 0.39 | 0.06 | 6.22 | <.001 |
| Stress | 0.13 | 0.05 | 2.35 | .02 |
| Age | -0.12 | 0.03 | -4.35 | <.001 |
| Male | 2.15 | 0.63 | 3.39 | <.001 |
| COVID-19 concern | -0.30 | 0.31 | -0.96 | .34 |
| Autistic traits x COVID-19 concern | 0.01 | 0.02 | 0.50 | .62 |
| Depression x COVID-19 concern | -0.01 | 0.04 | -0.15 | .88 |
| Anxiety x COVID-19 concern | -0.05 | 0.05 | -0.95 | .34 |
| Stress x COVID-19 concern | -0.02 | 0.05 | -0.49 | .62 |
| Age x COVID-19 concern | -0.01 | 0.02 | -0.58 | .56 |
| Male x COVID-19 concern | -0.96 | 0.58 | -1.65 | .10 |

*Note.* R^2^_adj_=.41

COVID-19 concern did not uniquely predict EDA traits and no interactions were statistically significant. Degree of concern about COVID-19 therefore did not modulate the pattern of results presented in the main paper.

**Supplementary References**

Brown, T. A., & Moore, M. T. (2012). Confirmatory factor analysis. In R. Hoyle (Ed.), *Handbook of structural equation modeling* (pp. 361–379). The Guilford Press.

Nelson, B. W., Pettitt, A., Flannery, J. E., & Allen, N. B. (2020). Rapid assessment of psychological and epidemiological correlates of COVID-19 concern, financial strain, and health-related behavior change in a large online sample. *PLOS ONE*, *15*(11), e0241990.

Rosseel, Y. (2012). lavaan: An R package for structural equation modeling. *Journal of Statistical Software*, *48*(i02).

Westfall, J., & Yarkoni, T. (2016). Statistically controlling for confounding constructs is harder than you think. *PloS One*, *11*(3), e0152719.
